# Simulation-free radiotherapy on the MR-linac in prostate cancer

**DOI:** 10.1093/bjr/tqaf163

**Published:** 2025-07-23

**Authors:** Sian Cooper, Joan Chick, Francis Casey, Sophie Alexander, Simeon Nill, Uwe Oelfke, Alison Tree, Alex Dunlop

**Affiliations:** The Institute of Cancer Research, London, SM2 5NG, United Kingdom; The Royal Marsden NHS Foundation Trust, London, SM2 5PT, United Kingdom; The Joint Department of Physics, The Royal Marsden Hospital and the Institute of Cancer Research, London, SM2 5PT, United Kingdom; The Joint Department of Physics, The Royal Marsden Hospital and the Institute of Cancer Research, London, SM2 5PT, United Kingdom; The Institute of Cancer Research, London, SM2 5NG, United Kingdom; The Royal Marsden NHS Foundation Trust, London, SM2 5PT, United Kingdom; The Joint Department of Physics, The Royal Marsden Hospital and the Institute of Cancer Research, London, SM2 5PT, United Kingdom; The Joint Department of Physics, The Royal Marsden Hospital and the Institute of Cancer Research, London, SM2 5PT, United Kingdom; The Institute of Cancer Research, London, SM2 5NG, United Kingdom; The Royal Marsden NHS Foundation Trust, London, SM2 5PT, United Kingdom; The Joint Department of Physics, The Royal Marsden Hospital and the Institute of Cancer Research, London, SM2 5PT, United Kingdom

**Keywords:** simulation free radiotherapy, SFR, simulation-free, prostate, MR-Linac, MRL, Unity

## Abstract

**Objectives:**

The radiotherapy (RT) pathway faces bottlenecks. The Rapid Adaptive and Cost-Effective Radiotherapy (RACE) study evaluates the feasibility of using diagnostic MRI (dMRI) scans for planning prostate MRI-guided adaptive RT (MRIgART).

**Methods:**

We audited prostate cancer patients treated with 5-fraction (#) stereotactic body radiotherapy (SBRT) between March 2023 and January 2024, assessing dMRI for RT planning suitability. Planning suitability required a T2-weighted sequence for target/organs at risk (OAR) delineation and a large field-of-view (LFOV). Scans were classified as RT plan suitable or as having specific issues (incomplete body coverage or slice thickness >10 mm). Workflow analysis from RT referral to first fraction estimated potential time savings with simulation-free RT (SFRT). Case studies illustrated identified issues and proposed solutions.

**Results:**

dMRIs were available for 93% of patients, with scans originating from various hospitals and conducted on 1.5 Tesla (T) or 3 T MRI scanners. Ideal image characteristics for RT planning were met in 38% of MRIs. Issues such as cropped field of view (FOV) and low slice resolution were identified, but proposed solutions could increase the number of patients with suitable scans to 87%.

**Conclusions:**

The findings suggest that with appropriate technical solutions, most dMRI scans can be adapted for RT planning purposes.

**Advances in knowledge:**

The study highlights the potential of SFRT to reduce treatment delays and improve cost-effectiveness.

## Introduction

Rising cancer incidence and resource constraints pressurize radiotherapy (RT) services, with bottlenecks in planning scan acquisition and the multidisciplinary planning process.^1^^,^^2^ SFRT addresses these challenges by leveraging prior dMRI instead of dedicated planning CT/MRI. For online adaptive radiotherapy (oART), daily imaging is used to adapt the reference plan, obviating the patient setup reproducibility requirement.

Emerging evidence from palliative RT demonstrates feasibility^3–5^ with reduced planning time and acceptable dosimetric accuracy.^6^ Its application to radical treatment using non-RT-dedicated dMRI remains underexplored, despite its potential benefits for the wider radiotherapy community.

### Aim

The RACE study evaluates the feasibility and safety of SFRT, specifically investigating whether non-RT-dedicated dMRI can serve as reference images for prostate MRIgART. This report presents preliminary findings: an audit of the availability of suitable dMRI for prostate RT and case studies illustrating a simulation-free (SF) workflow using dMRI reference planning. The RACE project is ongoing.

## Methods

### Diagnostic imaging audit methods

DMRI scans were audited from all prostate cancer patients who received 36.25 Gy/5# SBRT between March 2023 and January 2024 at our institution. RT planning suitability criteria were based on sequence parameters from DICOM data and field-of-view (FOV) coverage. dMRIs were RT plan suitable if they contained T2-weighted imaging for target/OAR delineation and a LFOV (of any contrast) scan for external body/bone contours. Patients with artificial hips were excluded. Within the “RT plan suitable” category, two issues were identified: “RT plan suitable—Issue 1” for scans where the FOV did not cover the entire extent of the body, and “RT plan suitable—Issue 2” for scans where the LFOV slice thickness was greater than 10 mm (see Table 1).

**Table 1.** Key parameters necessary for a diagnostic MR to be suitable for RT planning.

RT plan suitable	RT plan unsuitable
**Ideal scenario** T2w scan for outlining targets & OARs LFOV (T1w or T2w) scan to cover *entire* whole body extent **Issues** LFOV (T1w or T2w) scan covers majority of whole body (clipped lateral extent or anterior saturation band) LFOV scan low resolution in slice direction	No T2w scan LFOV scan does not cover majority of body Artificial hip(s)

RT referral and treatment dates were recorded. Intervals between workflow steps were measured to quantify potential SFRT time savings. Current workflow was measured from referral to first treatment, with SFRT savings calculated as the period between referral and planning MRI.

### SFRT case studies methods

Two patients (A and B) were selected for case studies. Both were treated on the 1.5 T MR-Linac (Unity, Elekta AB, Sweden) as part of the HERMES trial (NCT04595019).^7^ Each had prior dMRI available, including a 2D T1-weighted LFOV and a 2D T2-weighted small field-of-view (SFOV) image. Patient A’s LFOV included the full body outline, while Patient B’s LFOV was laterally cropped. Treatment datasets comprised CT and MR-Linac simulation scans, clinically approved reference plans based on MR-Linac imaging, and adapted plans generated using Monaco 5.40 (Elekta AB, Stockholm, Sweden). The CT scan provided patient-specific electron density (ED) and bone structure data.

A reference image (“SimFree”) was generated from dMRI by upsampling to 1 mm resolution using linear interpolation and correcting image tilt (MATLAB version 9.12, R2022a, The MathWorks Inc., Natick, MA, United States). Target volumes and OARs were contoured on the T2-weighted SFOV, while bones and body were segmented on the fused T1-weighted LFOV (RayStation, version 2024B, RaySearch Laboratories AB, Stockholm, Sweden). For patient B, the body contour was manually estimated, extended, and adjusted with an intensity mask to match the body contour (MATLAB). A synthetic CT was generated via bulk density assignment, applying population-average ED to body, bone, and target structures.^8^^,^^9^ MR-Linac reference plans were created on SimFree images using Monaco (v6.2.1), following the HERMES trial protocol.^7^ Daily plan adaptation utilized clinical target/OAR volumes, with body and bone contours deformably propagated from dMRI. SimFree-adapted plans were compared to clinical plans to assess compliance with clinical goals.

## Results

### Diagnostic imaging audit results

dMRI was available for 39/42 (93%) patients identified from 13 hospitals, with scans performed on 1.5 Tesla (T) (79%) and 3 T (21%) systems, primarily Siemens (Siemens AG, Munich, Germany) (69%) and GE scanners (General Electric, Boston, MA, United States). All images underwent scanner-applied distortion correction (mean bandwidth: 259 Hz/mm, range 98-816) and included SFOV 2D T2-weighted sequences (slice separation: 3-3.9 mm, one outlier at 6.25 mm) and LFOV 2D T1-weighted sequences (2-10.4 mm separation). 33% of examinations included an LFOV 2D T2w.

Of the 39 patients with available dMRI, 87% (34/39) had scans suitable for RT planning. Among these, 38% (15/39) met all ideal criteria, while 31% (12/39) had minor issues such as cropped lateral extents (issue 1) or anterior saturation bands on the LFOV. An additional 18% (7/39) had low slice resolution (>10 mm, issue 2) on the LFOV. Only 13% (5/39) of scans were completely unsuitable for planning, mainly due to unresolvable issues such as artificial hips or severely limited FOV.

**Figure 1.**
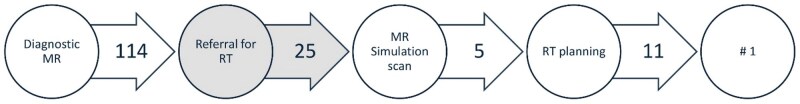
Timeline for pre-treatment workflow. Numbers in arrows indicate the median number of calendar days between timepoints for the patients in the study, with the shaded shapes representing potential days saved with simulation-free workflow. # = Fraction.

The median time between dMRI and the start of treatment was 157 days (range from 78 to 353). By adopting an SF workflow, the treatment pathway could potentially be shortened by a median of 25 days (Table 2, Figure 1).

**Table 2.** Time for each stage of the pre-treatment workflow. The current workflow is represented by “RT referral and #1” and simulation-free represented by “RT referral and MR simulation scan.”

	Pre-treatment steps				Time between key pathway points		
Time point (days between)	Diagnostic MR and referral for RT	RT referral and MR simulation scan	MR simulation scan and RT planning	RT planning and #1	Diagnostic MR and #1	RT referral and #1	MR simulation scan and #1
Median (range)	114 (49-292)	25 (5-78)	5 (1-65)	11 (5-26)	157 (78-353)	40 (18-103)	16 (8-42)

### SFRT case studies results

**Figure 2.**
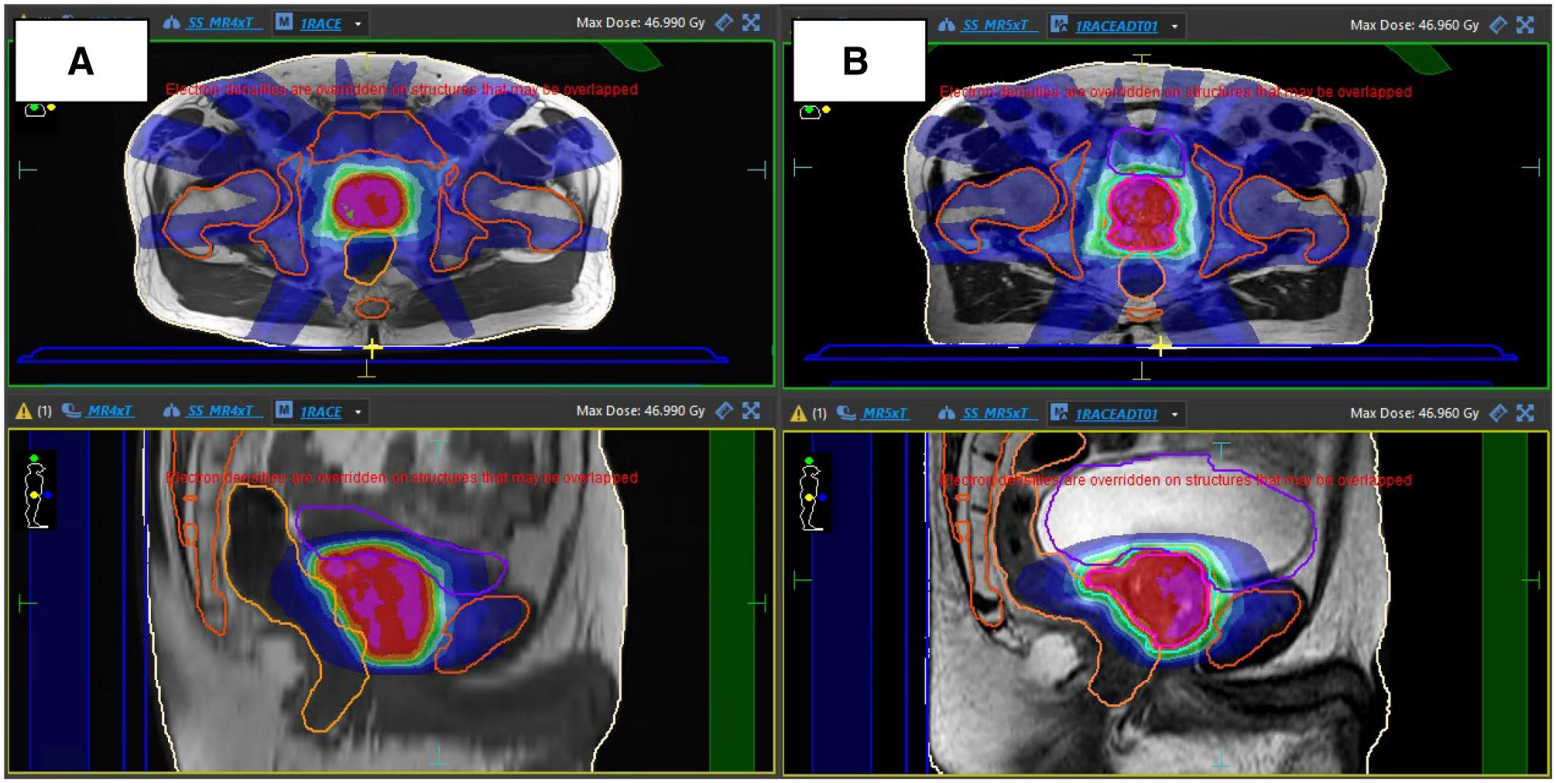
**(A)** An example of a reference prostate RT plan on a diagnostic LFOV T1w, illustrating a non-flat tabletop and a small bladder reflecting no specific organ preparation guidelines. **(B)** An example of the adapted plan on an MRL image, with deformed bones and body, with the clinically defined targets and OARs.

**Figure 3.**
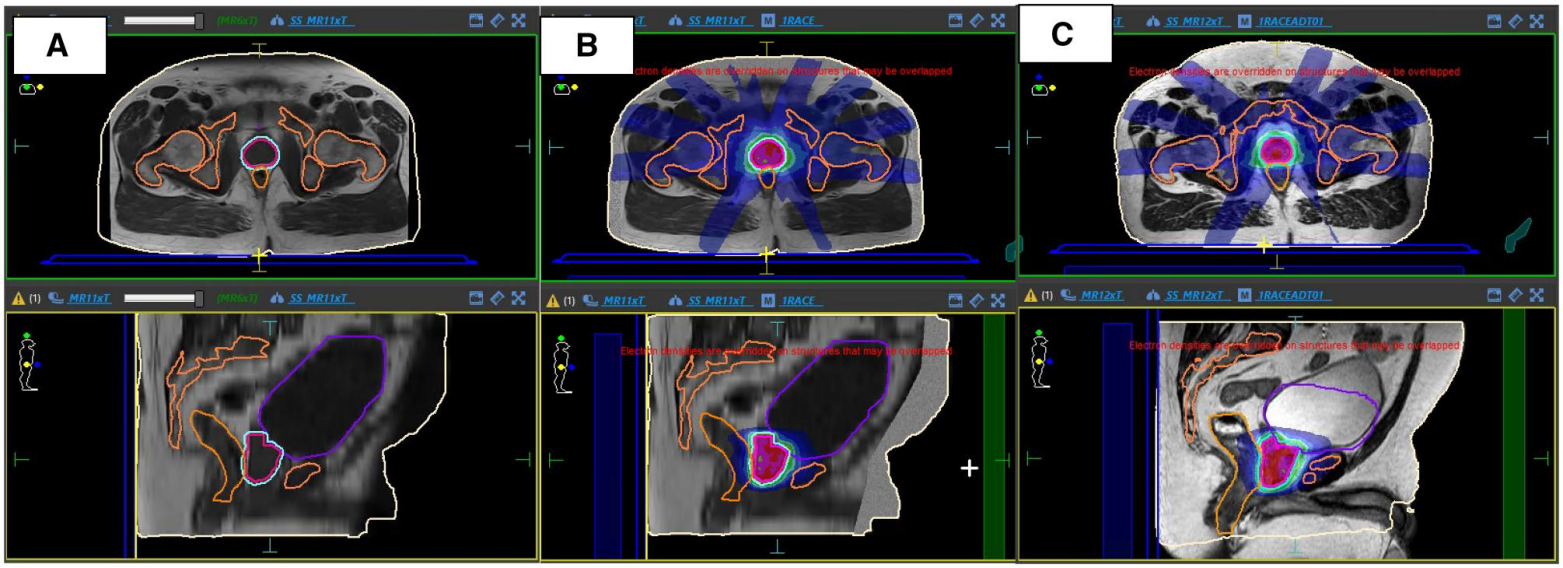
**(A)** An example of a diagnostic LFOV T1w, with missing a body outline (Issue 1). **(B)** An example of a simulated image with a full-body estimate and image masks used to simulate the image intensity, with a reference plan. **(C)** An example of the adapted plan on an MRL image, with deformed bones and body, with the clinically defined targets and OARs.

SimFree reference images and treatment plans were successfully created for both patients, meeting all clinical goals. We also generated adapted plans by deformable propagation of bone and body contours from the diagnostic SimFree images to daily MR-Linac images. These adapted plans satisfied all clinical requirements. Figure 2 illustrates patient A’s workflow, showing both reference and adapted plans. For Patient B, Figure 3 displays the original dMRI, the SimFree reference plan, and the adapted plan. These results confirm that dMRI can serve as a reference for clinically acceptable radiotherapy plans without conventional simulation.

## Discussion

This preliminary work demonstrates that most diagnostic MRIs from diverse referral sources can be repurposed for radical RT planning despite scanner protocol variations and technical limitations. Only 13% of scans were unsuitable due to unresolvable issues like limited FOV or artifacts.

We propose generating oART reference plans by fusing diagnostic LFOV scans with SFOV T2-weighted images for target and OAR contouring. Daily adaptation on MR-Linac systems mitigates challenges: non-flat diagnostic MRI couches (Figure 2), absence of bladder/bowel preparation, and spatial distortions from lower-bandwidth dMRIs. While only 35% of dMRIs met IPEM guidelines for receiver bandwidth (>225 Hz/mm for 1.5 T and >450 Hz/mm for 3 T,^10^ high-bandwidth 3D MR-Linac daily imaging ensures minimal distortion in adapted treatment plans. Deformable registration (Figure 2) effectively addresses anatomical changes and residual distortions from low-bandwidth diagnostic scans.

**Figure 4.**
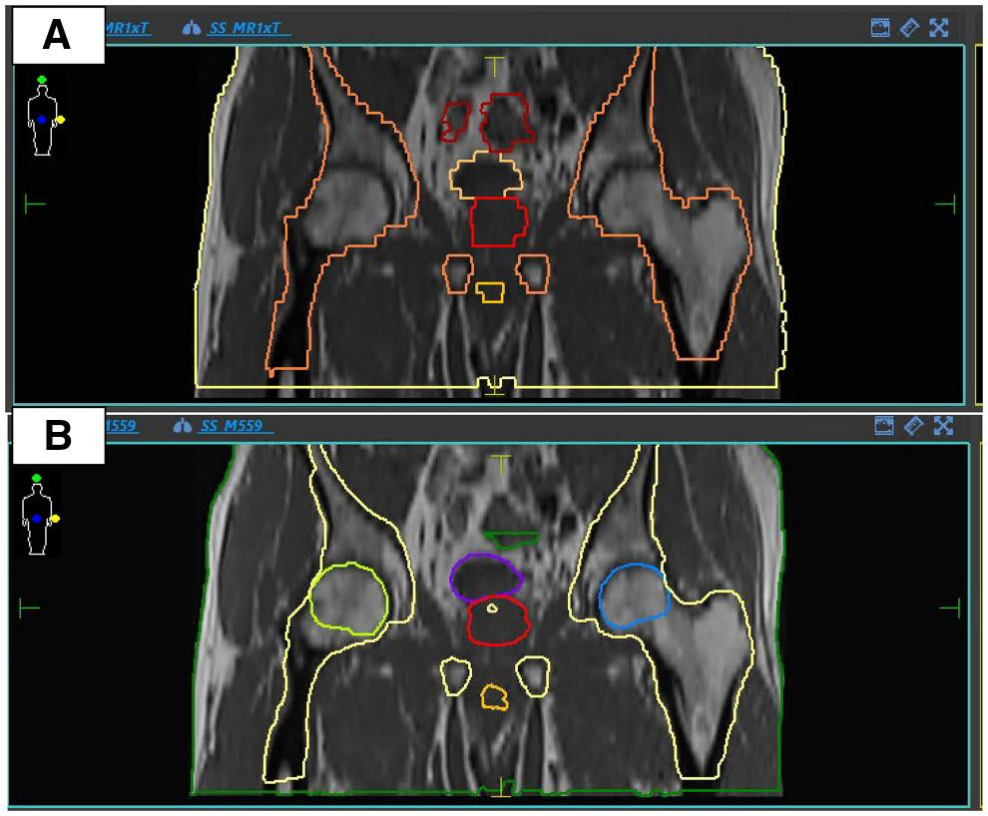
**(A)** Example of a coronal view of a diagnostic image with >10mm slice thickness (Issue 2), showing the ‘stepped’ structures as outlined on the axial slices. **(B)** Example of a coronal view of the same data set interpolated to 1 mm slice thickness before outlining. The structures now are smooth and reflect the underlying 3D anatomy. The underlying image is interpolated for visualization.

**Figure 5.**
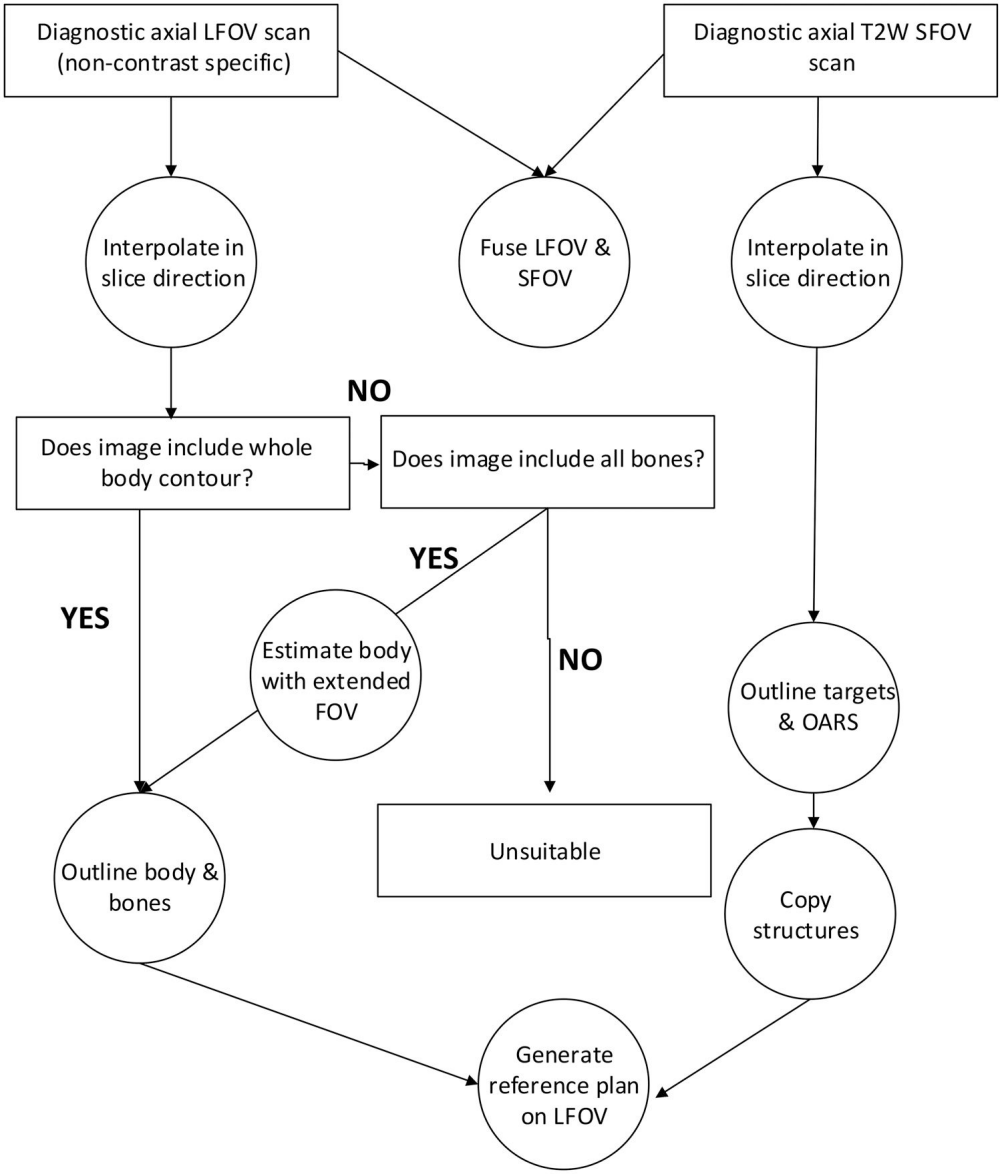
Flow chart for generating reference plan using diagnostic MRI.

The case study methods successfully addressed audit-identified challenges, producing clinically acceptable plans. For low spatial resolution in LFOV dMRI (slice separation 2-10.4 mm), interpolation provided the solution, preventing pixelated reference structures from being deformed onto daily MR-Linac images (Figure 4). Lateral extension techniques resolved cropped body outlines (FOV 260-400 mm), while the MR-Linac’s larger 480 mm FOV during treatment further mitigates uncertainties in body outline. A suggested workflow is shown in Figure 5.

This workflow represents only the first step in the ongoing RACE project. Comprehensive validation of its accuracy and safety will be addressed in future phases and publications.

SFRT is especially valuable for patients with travel challenges, reducing pre-treatment visits. A New South Wales study using AI-assisted adaptive workflows reported 20-minute adaptation times, though it focused on palliative RT, unlike our radical approach.^3^

Our audit indicates that SFRT could reduce the pre-treatment workflow by over three weeks, offering a significant advantage for time-sensitive cases.^11^ However, published data show mixed results. In a comparative study of palliative SFRT and conventional CT-based workflows, SFRT reduced median plan generation time (0.88 vs. 1.90 days, *P* = .02), but the absolute difference was modest and total treatment time was actually longer for SFRT (41 vs. 30 minutes, *P* = .02), though dosimetry remained clinically acceptable.^5^ Centers with longer simulation delays may see greater benefit.

The ongoing phase II DART trial is also evaluating diagnostic CT-enabled planning versus conventional CT simulation, with patient time in the cancer center as the primary endpoint.^12^ Early results show a substantial reduction in time for palliative patients (median 4.7 to 0.41 hours), though this palliative, two-field approach differs from our radical adaptive workflow.

There are limitations to our current work. This single-center study focuses on MRIgART for prostate cancer; well-suited for SFRT due to stable anatomy. Techniques may be adapted to other platforms, but rapidly changing tumors or post-surgical cases require current imaging. Complete body outlines may not always be captured, especially in larger patients.

Despite these limitations, this approach represents a paradigm shift in radiotherapy delivery. By leveraging existing diagnostic imaging and adaptive planning, it has the potential to improve treatment effectiveness, reduce costs, and minimize patient anxiety associated with treatment delays. Ongoing research is focused on finding the optimal balance between time efficiency and plan quality when using diagnostic scans for online adaptive radiotherapy.

## Conclusion

Most dMRIs are suitable for MRIgART prostate planning, and identified challenges can be addressed with technical solutions.
